# Supplementary material for: Spatial distribution modulation of mixed building blocks in metal-organic frameworks
Source: Nat Commun. 2022 Feb 24;13:1027. doi: 10.1038/s41467-022-28679-w (PMC8873209; doi:10.1038/s41467-022-28679-w)
Supplement: Supplementary file 1 — Supplementary Information [file 41467_2022_28679_MOESM1_ESM.pdf]

Supplementary information

Spatial distribution modulation of mixed building blocks in metal−organic frameworks

Seok Jeong[1], Junmo Seong[1], Sung Wook Moon[1], Jaewoong Lim[1], Seung Bin Baek[1], Seung Kyu Min[1*] & Myoung Soo Lah[1*]

Department of Chemistry, Ulsan National Institute of Science and Technology, Ulsan 44919, Korea

E-mail: skmin@unist.ac.kr; mslah@unist.ac.kr

Computational details

Density functional theory calculation of energy difference between HAP and HBE. Energy difference between reactants (HAP crystal with unligated BE pillars) and products (HBE crystal with unligated AP pillars) was calculated using spin-polarized density functional theory (DFT) employing Vienna ab initio simulation package (VASP)[1] with Perdew-Burke-Ernzerhof (PBE) functional[2] and Grimme-D3 dispersion corrections[3]. A 2×2×2 k-point sampling with a 400 eV kinetic energy cutoff was adopted. The geometry and unit cell parameters of HAP (or HBE) model structures containing 6 AP (or BE) pillars obtained from HAP crystal were optimized before energy calculations. Since the pillar exchange reaction deals with HAP crystals with entering BE and leaving AP pillars, the energy difference (ΔE') between the reactants and products of the pillar exchange reaction is defined as E'(HBE) – E'(HAP). Total energy E'(HAP) of an HAP crystal with 6 BE pillars in the unit cell is (E_{HAP} + 6E_{BE})/6, where E_{HAP} is the energy of the HAP crystal and E_{BE} is the energy of an isolated entering BE pillar. E_{BE} was also calculated after geometry optimization with a supercell at a Γ-point with a 400 eV kinetic energy cutoff. Total energy E'(HBE) is defined in the same way for E'(HAP), but with E_{HBE} and E_{AP} instead of E_{HAP} and E_{BE}. The calculated energy difference ΔE′ between the reactants and products is –11.7 kJ/mol.

Kinetic Monte Carlo simulation. For the kinetic Monte Carlo (kMC) simulation, a two-dimensional triangular cell with a total of 378 grid points is built as a 3D framework model of HAP/HBE to mimic realistic molecular situations. Each side of the triangular cell consists of 27 grid points corresponding to L = 27 nm. The triangular cell is further expanded into a hexagonal cell system by applying a periodic boundary condition, where the two edges of a triangular cell face the symmetry-related edges of the adjacent triangular cells, and the other remaining edge serves as the diffusion boundary of the free

pillar. The pillar exchange process is assumed to occur equivalently for each layer. The "reservoir effect" is considered by renormalizing the number of free pillars at the boundary. For forward/reverse pillar exchange, inward diffusion of the free pillar *trans*-1,2-bis(4-pyridyl)ethene (BE)/azobis(4-pyridine) (AP) from the reservoir into the hexagonal cell system occurs via renormalization of the number concentration of BE/AP at the boundary. On the other hand, outward diffusion of the free pillar AP/BE occurs via removal of the free pillars AP/BE from the boundary. To efficiently describe the exchange and diffusion of pillars, the grid is set as a dual grid comprising the exchange grid (EG) and diffusion grid (DG). Each EG point can have two different exchange states, MAP and MBE, where MAP and MBE represent the state of an AP ligated to a metal ion (M–AP) and that of a BE ligated to a metal ion (M–BE), respectively. Meanwhile, each DG point can have three different diffusion states (0, AP, and BE): the absence of both AP and BE (i.e., the vacancy of the sites intended for occupation by pillars or the presence of solvent alone), the presence of AP, and the presence of BE. Therefore, each dual grid point can have six different |E,D> states: |MAP,0>, |MAP,AP>, |MAP,BE>, |MBE,0>, |MBE,AP>, and |MBE,BE>, where |E,D> represents the exchange and diffusion states of each dual grid point.

The following are the three possible events that can occur during the pillar exchange process: forward exchange reaction, |MAP,BE> → |MBP,AP>; reverse exchange reaction, |MBE,AP> → |MAP,BE>; pillar diffusion to the nearest grid point, |E,AP> (or |E,BP>) + |E,0>$_{NN}$ → |E,0> + |E,AP>$_{NN}$ (or |E,BE>), where NN represents one of the nearest neighboring grid points. The exchange event at an EG point can occur only if two different pillars are simultaneously present at both EG and DG, while the diffusion event from a DG point to the nearest grid point can happen only if there is at least more than one vacancy at

the grid points nearest to the DG point. The triangular cell has six nearest grid points. Initially, all the grid points for the forward (or reverse) exchange reactions are set at |MAP,0> (or |MBE,0>), that is, all the EG points are filled with MAP (or MBE) and all the DG points are filled with solvent molecules. When the energy barrier of the reverse pillar exchange is considerably higher than that of the forward process, the entering pillar ligated to an EG point will not be re-exchanged with the leaving pillar at the DG point because the free leaving pillar can easily smear out from the DG point. On the other hand, when the energy barrier of the reverse pillar exchange is considerably lower than that of the forward process, the entering pillar ligated to an EG point can be re-exchanged with the free leaving pillar at the DG point because the free leaving pillar does not smear out or partially smears out from the DG point. To account for the behavior of free leaving pillar after pillar exchange through the forward and reverse pillar exchange processes, the effect of the "stay" probability, ρ, on spatial pillar distribution was investigated, where ρ is defined as the probability of a free leaving pillar remaining at a DG point without spreading toward the nearest vacant neighboring DG points on the reservoir side of the hexagonal grid cell, which is introduced to investigate the chemical potential gradients of the leaving pillars.

The rate constants for forward/reverse pillar exchanges and pillar diffusions are defined as k_f/k_r and k_d, respectively, where the temperature dependence of k_f/k_r is computed using the Arrhenius equation with the same pre-exponential factor (A). The energy barriers for the forward and reverse exchanges are set at 21 kcal/mol and 23 kcal/mol, respectively, to account for the experimental results. The diffusion rate constant of the free pillar from a DG point to one of the nearest neighboring DG points is set to $10^{-14}A$, that is, 10^{-14} diffusion events occur per unit time ($1/A$). The temperature dependence of pillar diffusion is ignored based on the assumption that the pillar diffusion barrier is considerably smaller than the

exchange reaction barrier. The parameter sets used for kMC simulations at different temperatures are listed in Supplementary Table 1. According to the typical kMC algorithm, the probability k_i/k_t for the event *i* is calculated, where k_i is the rate constant for the event *i* at a given time, and k_t is the sum of all possible rate constants. The grid point where the event will occur is selected from a uniform random number in [0,1], and the grid point information is updated. The duration of each event is determined by $\Delta t = -\ln(r)/k_t$, where *r* is another uniform random number in [0,1]. The MonteCoffee code[4] has been modified to apply the algorithm mentioned above. The snapshot of the mean spatial pillar distributions $D_{MAP/MBE}(x,t;T)$ of MAP and MBE with a "stay" probability ρ was obtained from 20 independent kMC simulations at 0, 50, 100, and 150 °C at given times. The "stay" probability ρ is defined as the probability of a free leaving pillar staying at a DG point without spreading toward the nearest vacant neighboring DG points on the reservoir side of the hexagonal grid cell, which is introduced to investigate the chemical potential gradients of the leaving pillars. $D_{MAP/MBE}(x,t;T)$ is calculated as $N_{MAP/MBE}(x,t;T)/N(x)$ (where $N_{MAP/MBE}$ is the number of grid points of MAP/MBE as a function of a radial factor $x = (L–R)/L$ at a normalized time *t* and a given temperature *T*, *N* represents the sum of the ligated pillars numbered N_{MAP} and N_{MBE}, and *L* and *R* are the edge length of the hexagonal grid cell and the distance between the center of the hexagonal grid cell and the position of MAP/MBE in the cell, respectively).

Supplementary Table 1. Rate constants and diffusion coefficients used in kinetic Monte Carlo simulations.

T (°C)	0	50	100	150
k_f, M–AP → M–BE (A)	1.00×10^{-17}	4.55×10^{-15}	3.95×10^{-13}	1.18×10^{-11}
k_r, M–BE → M–AP (A)	2.42×10^{-19}	1.96×10^{-16}	2.60×10^{-14}	1.08×10^{-12}
k_d, diffusion (A)	1.00×10^{-14}	1.00×10^{-14}	1.00×10^{-14}	1.00×10^{-14}

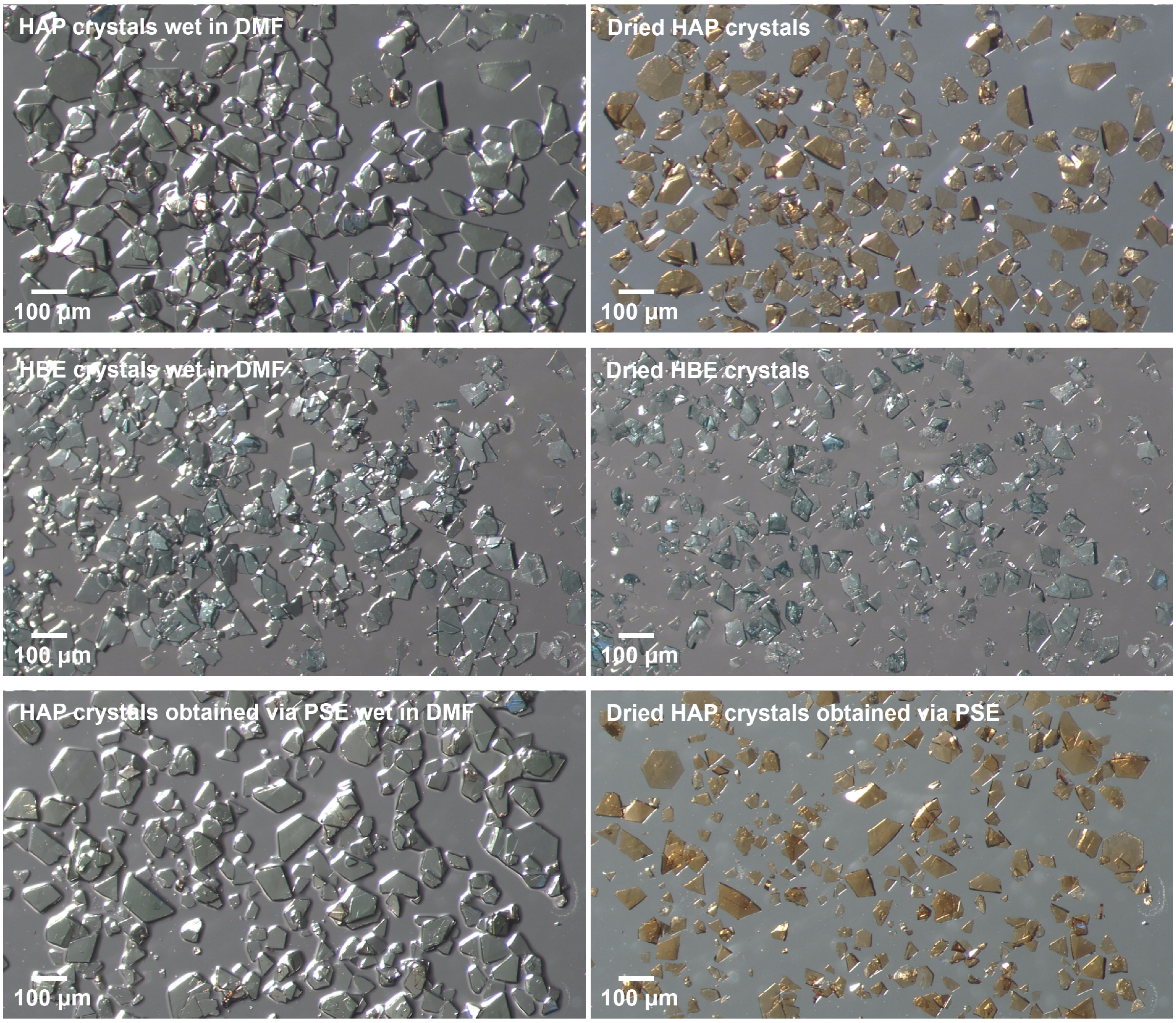

Supplementary Figure 1. Optical photographs of HAP single crystals, HBE single crystals, and HAP single crystals obtained through PSE, washed with DMF, and dried under ambient conditions for 2 h.

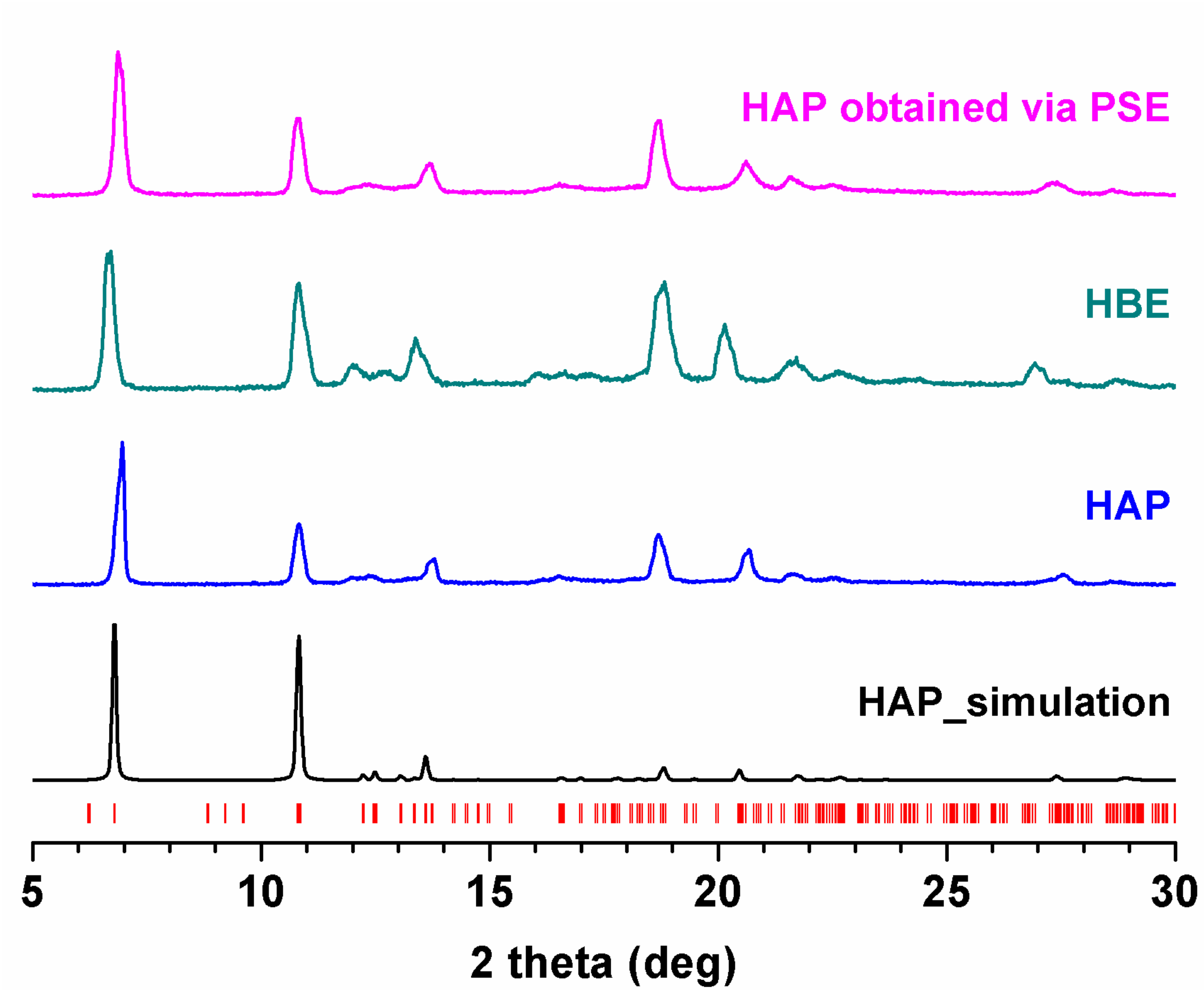

Supplementary Figure 2. PXRD patterns of HAP, HBE, and HAP obtained through PSE.

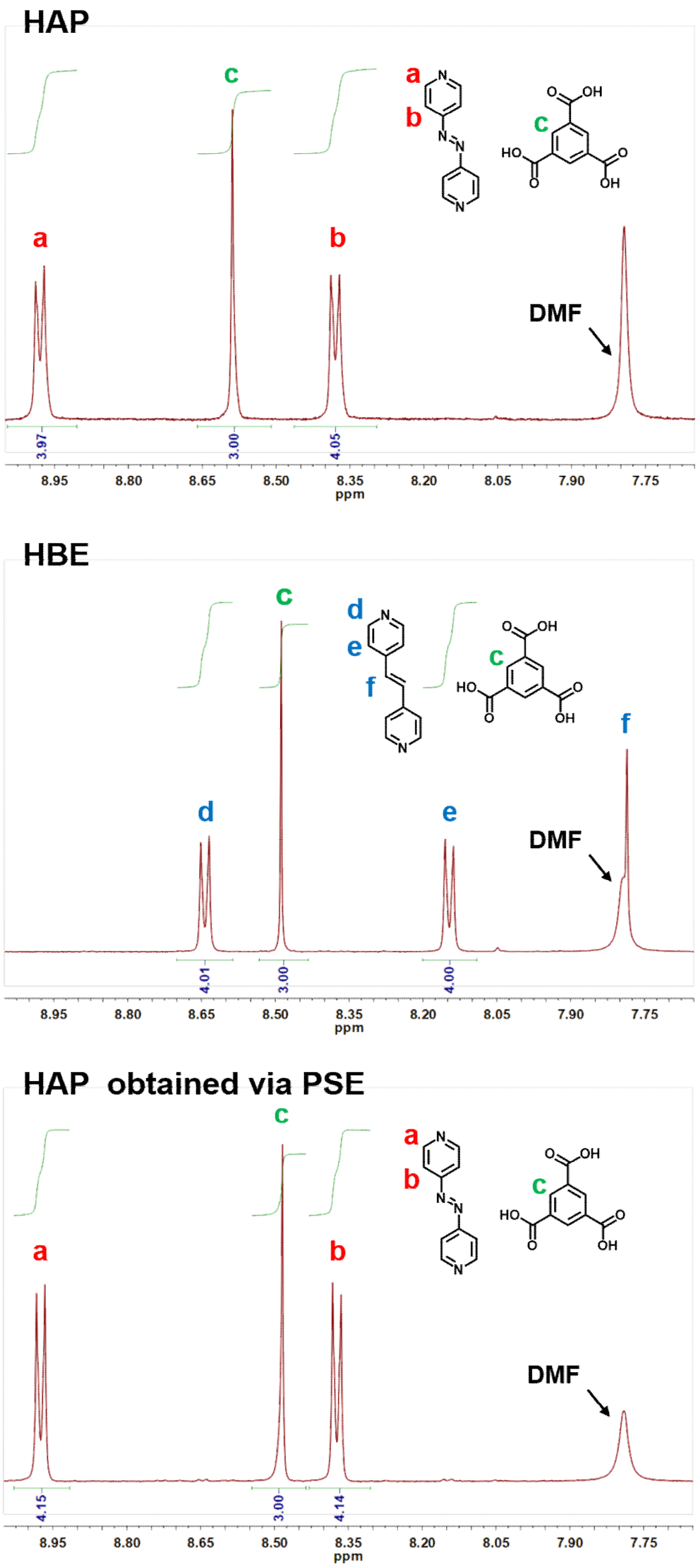

Supplementary Figure 3. ^{1}H NMR spectra of HAP, HBE, and HAP obtained through PSE and digested in $DCl/D_2O/DMSO$-d_6.

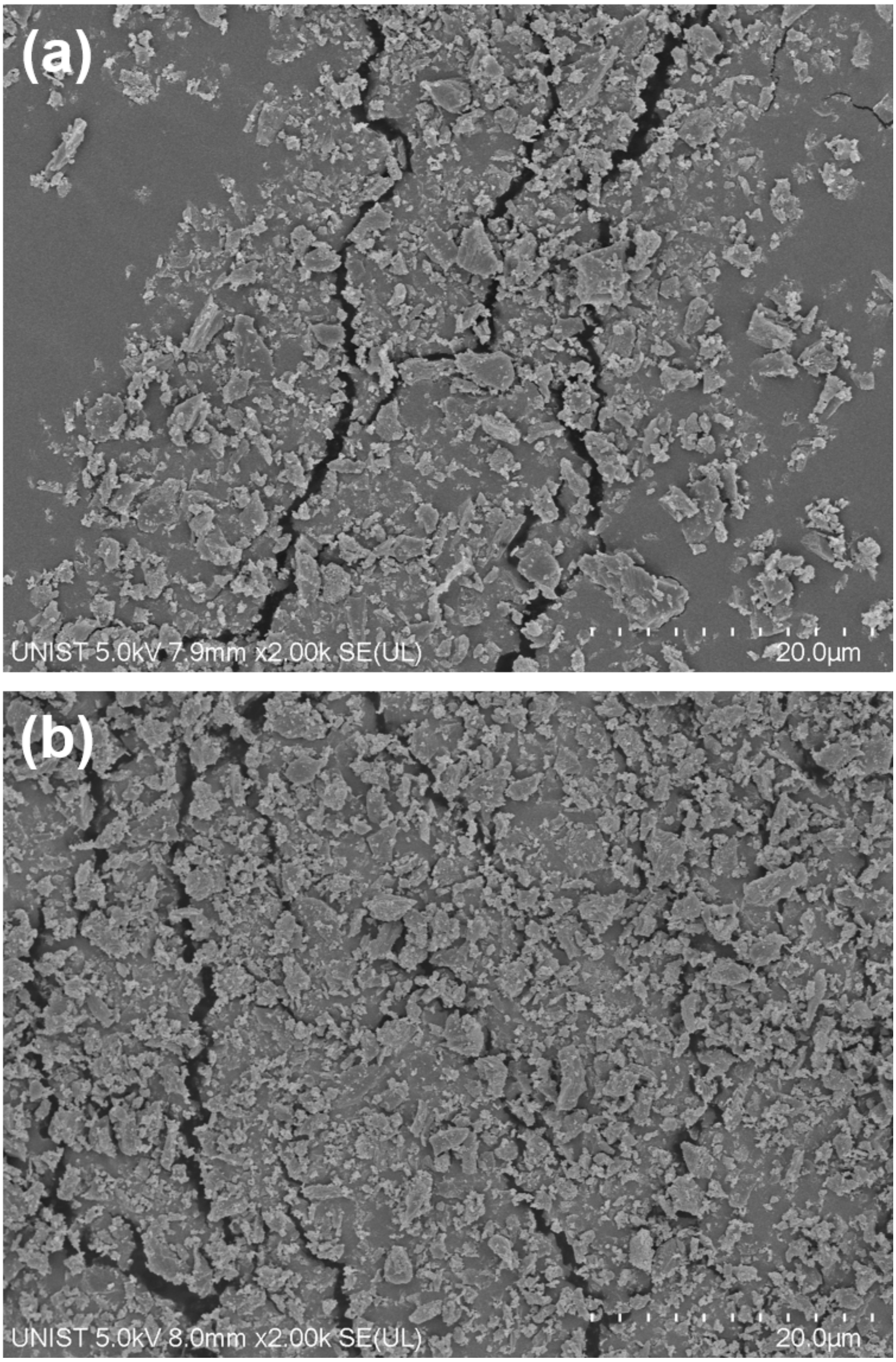

Supplementary Figure 4. SEM images of wet-ground (a) HAP (b) and HBE crystals.

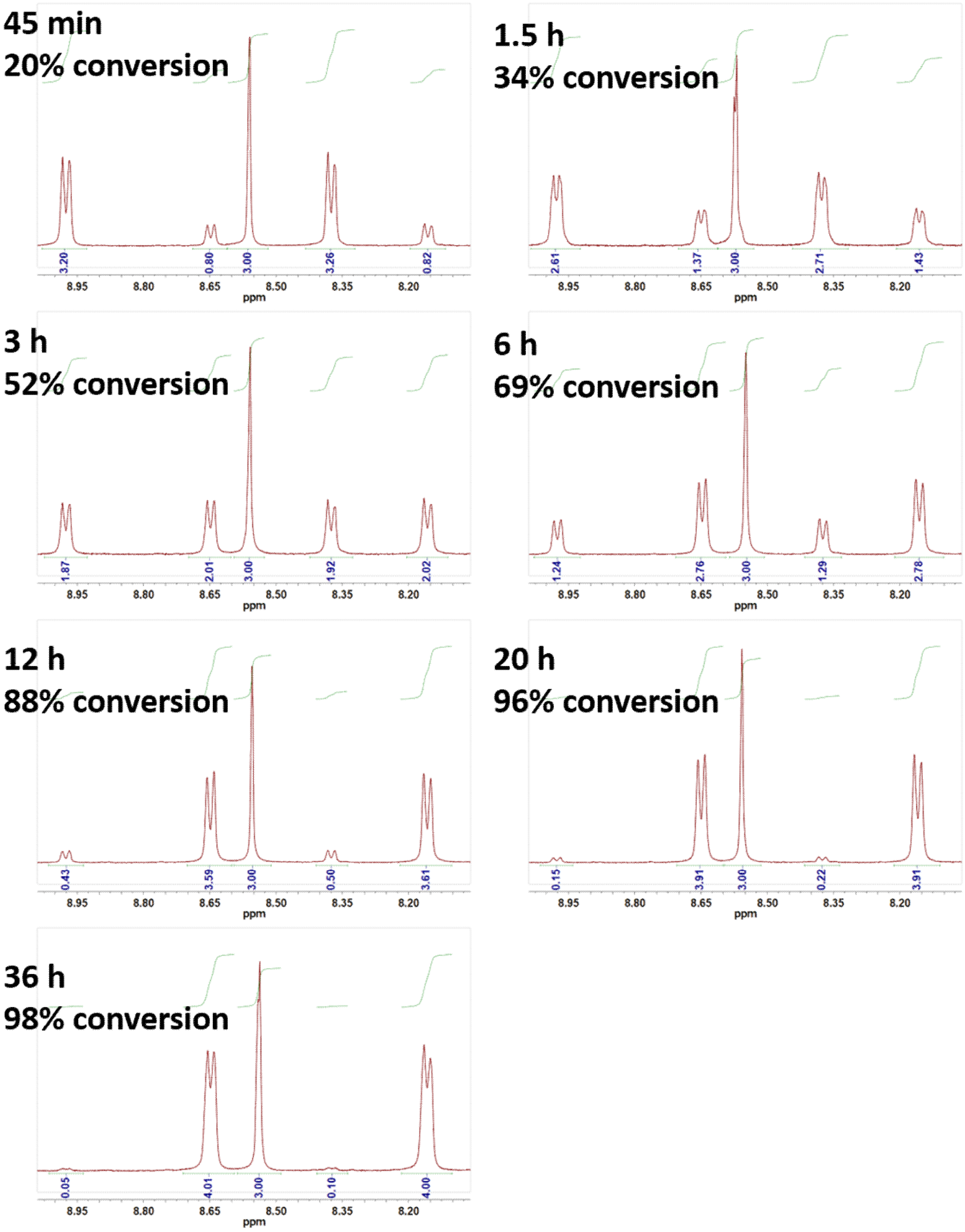

Supplementary Figure 5. ^{1}H NMR spectra of approximately 2 mg of well-ground HAP crystals soaked in 1.5 mL 0.3 M BE-DMF solution at 30 °C for given times.

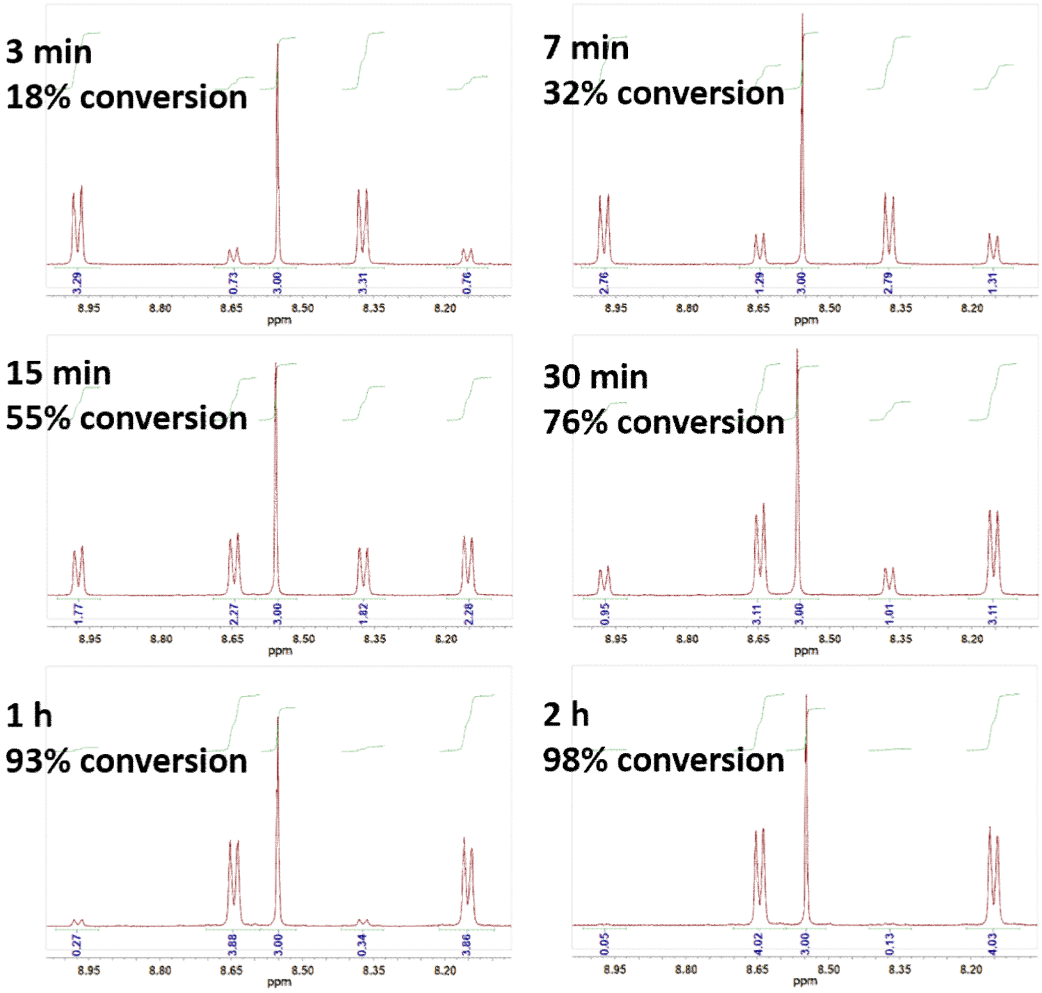

Supplementary Figure 6. ^{1}H NMR spectra of approximately 2 mg of well-ground HAP crystals soaked in 1.5 mL 0.3 M BE-DMF solution at 50 °C for given times.

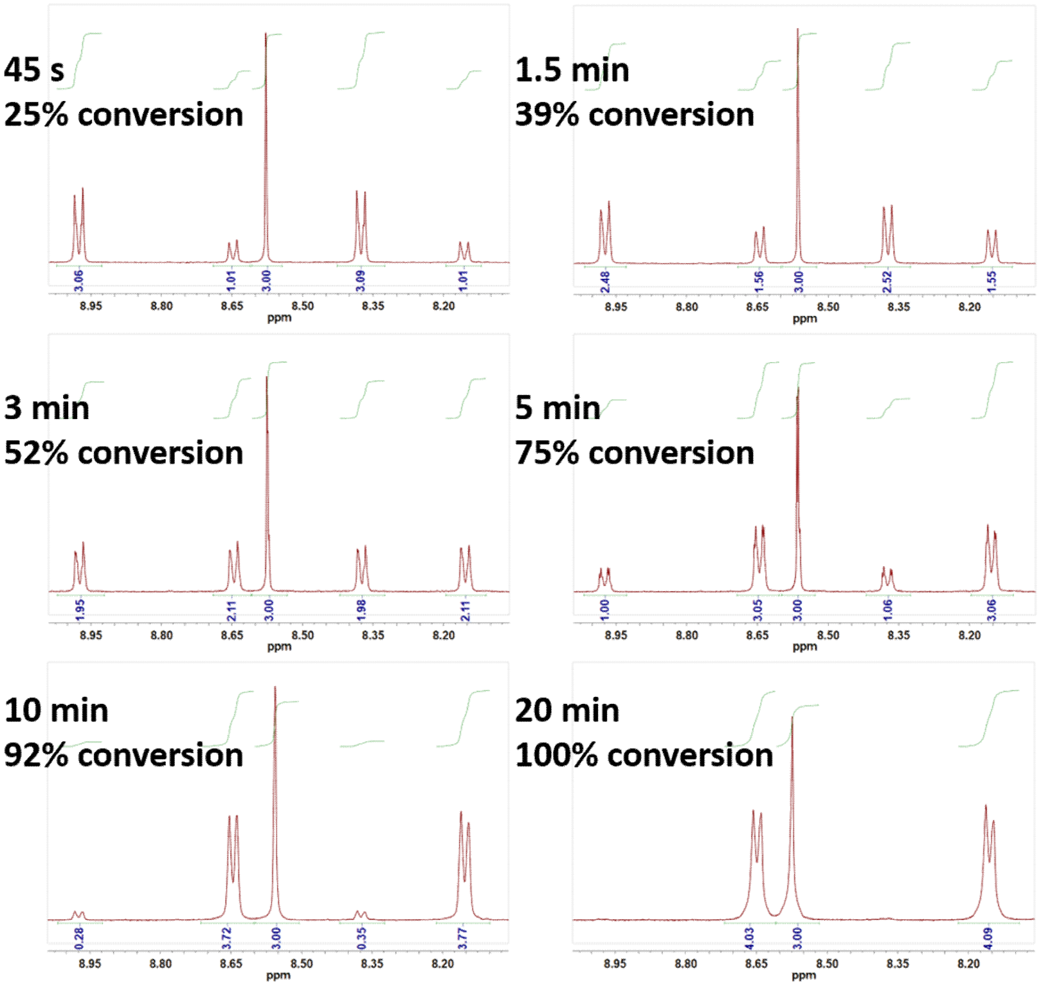

Supplementary Figure 7. ^{1}H NMR spectra of approximately 2 mg of well-ground HAP crystals soaked in 1.5 mL 0.3 M BE-DMF solution at 70 °C for given times.

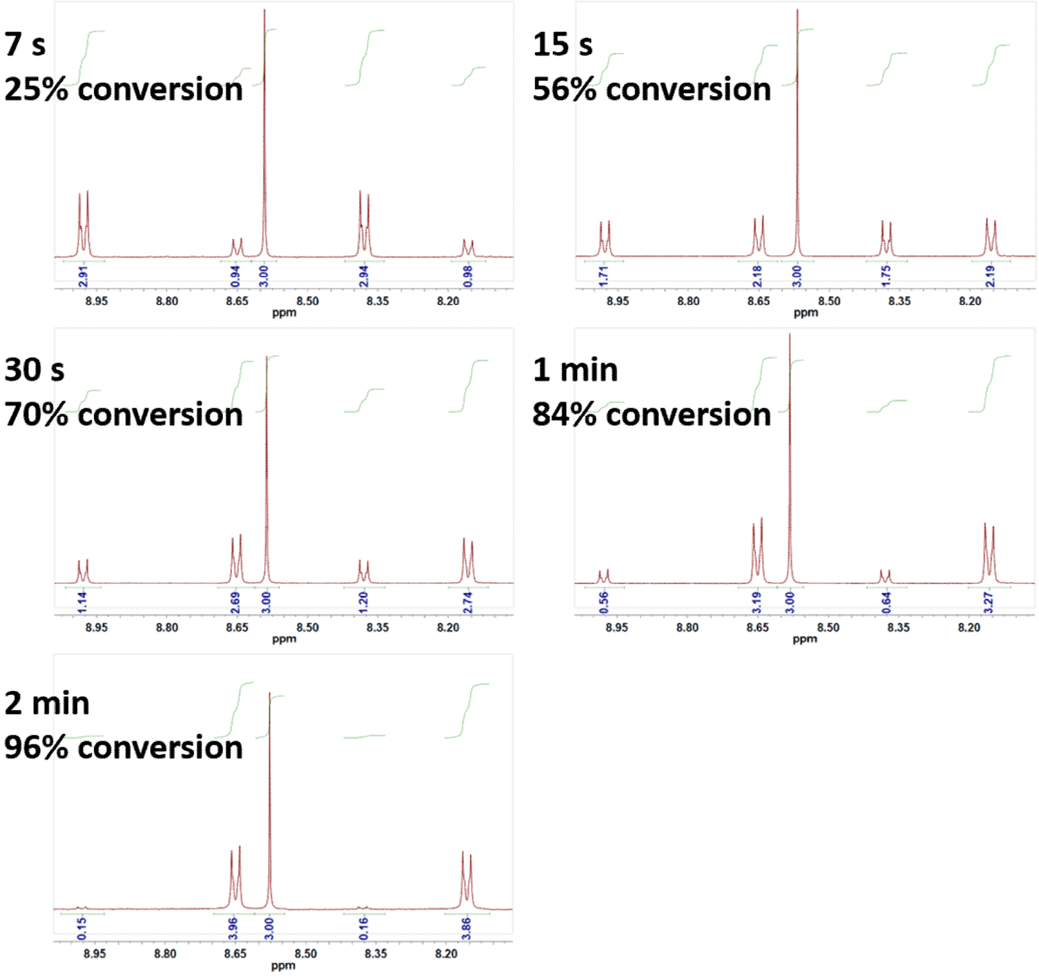

Supplementary Figure 8. ^{1}H NMR spectra of approximately 2 mg of well-ground HAP crystals soaked in 1.5 mL 0.3 M BE-DMF solution at 100 °C for given times.

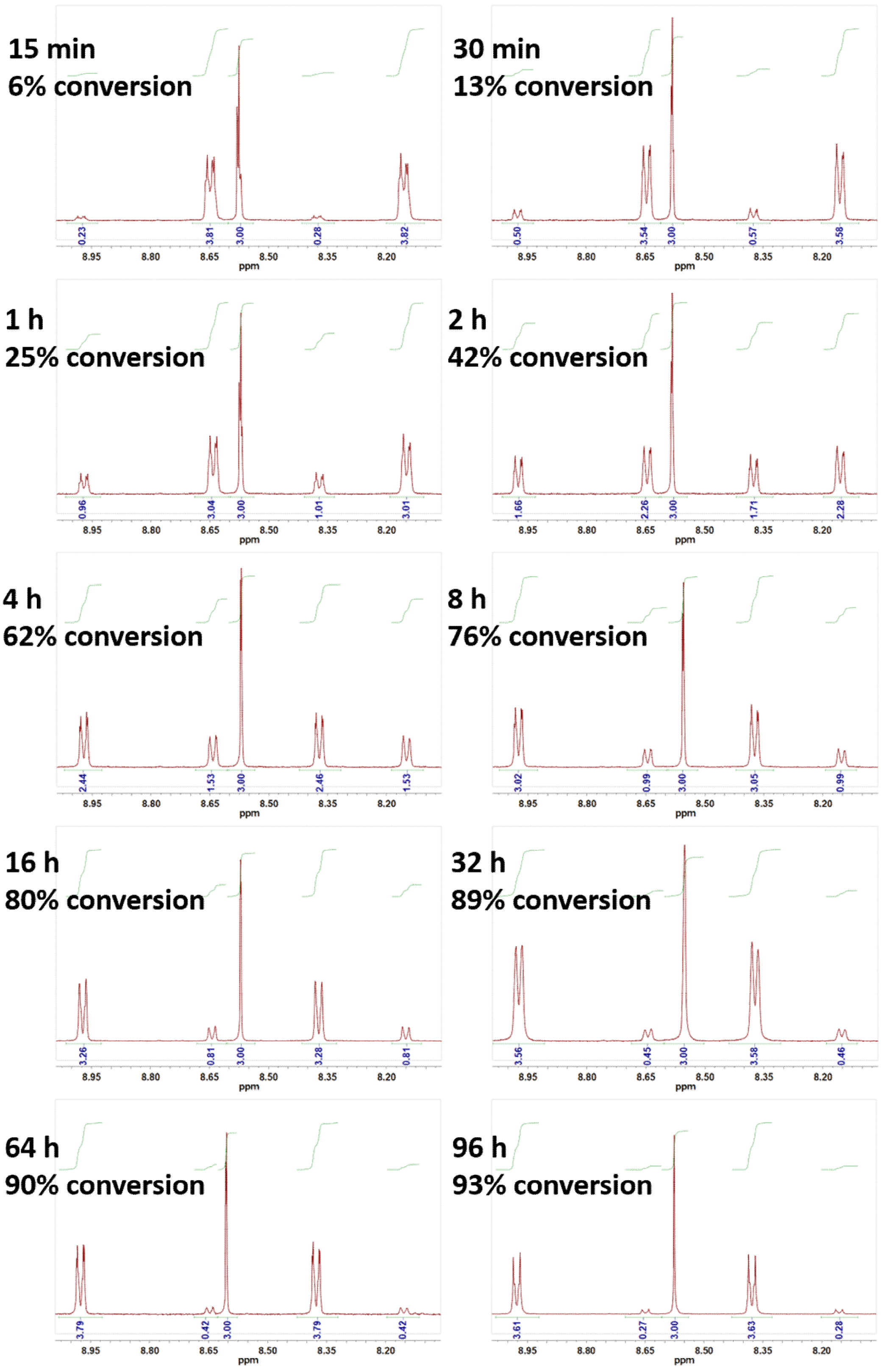

Supplementary Figure 9. ^{1}H NMR spectra of approximately 2 mg of well-ground HBE crystals soaked in 1.5 mL 0.3 M AP-DMF solution at 50 °C for given times.

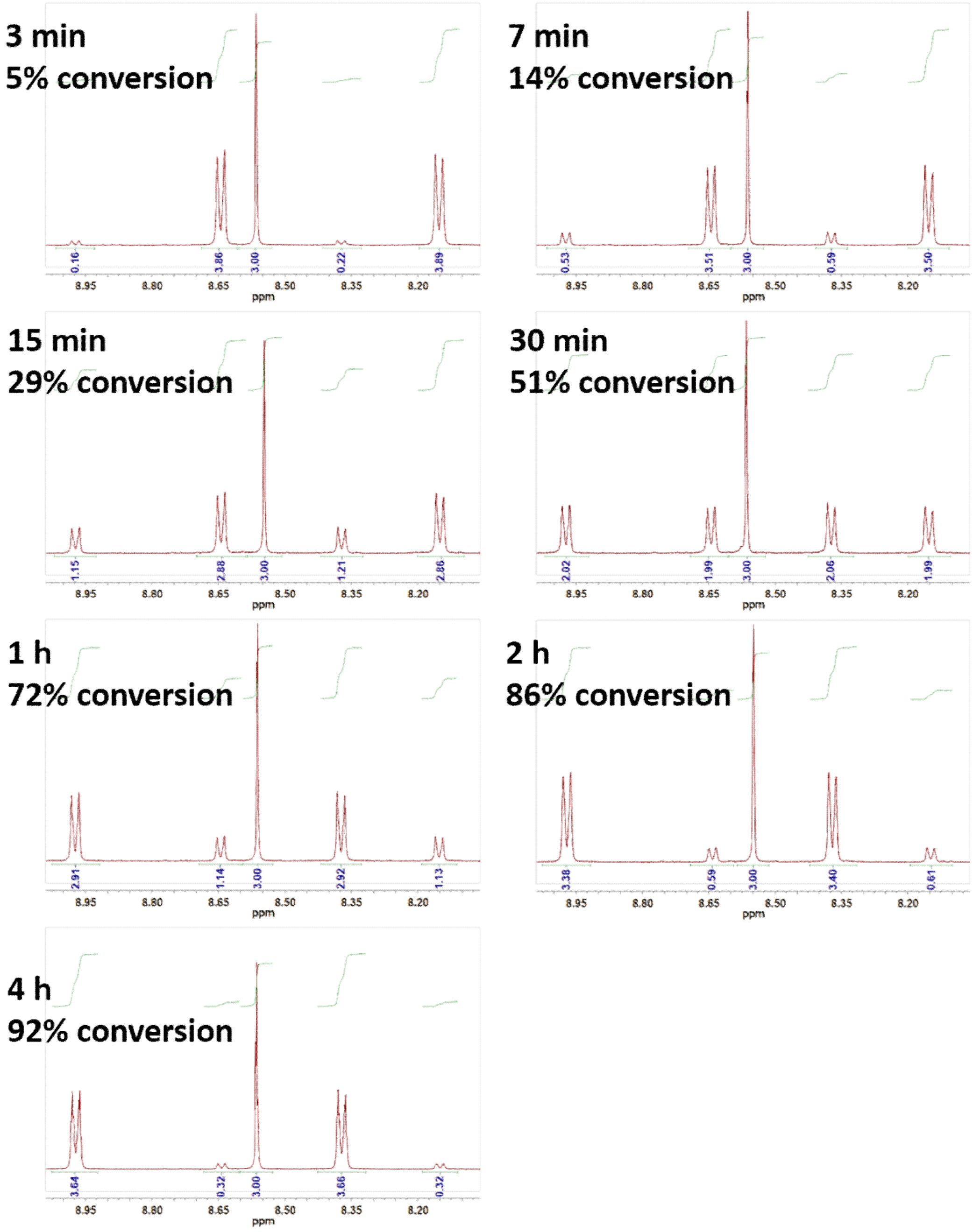

Supplementary Figure 10. ^{1}H NMR spectra of approximately 2 mg of well-ground HBE crystals soaked in 1.5 mL 0.3 M AP-DMF solution at 70 °C for given times.

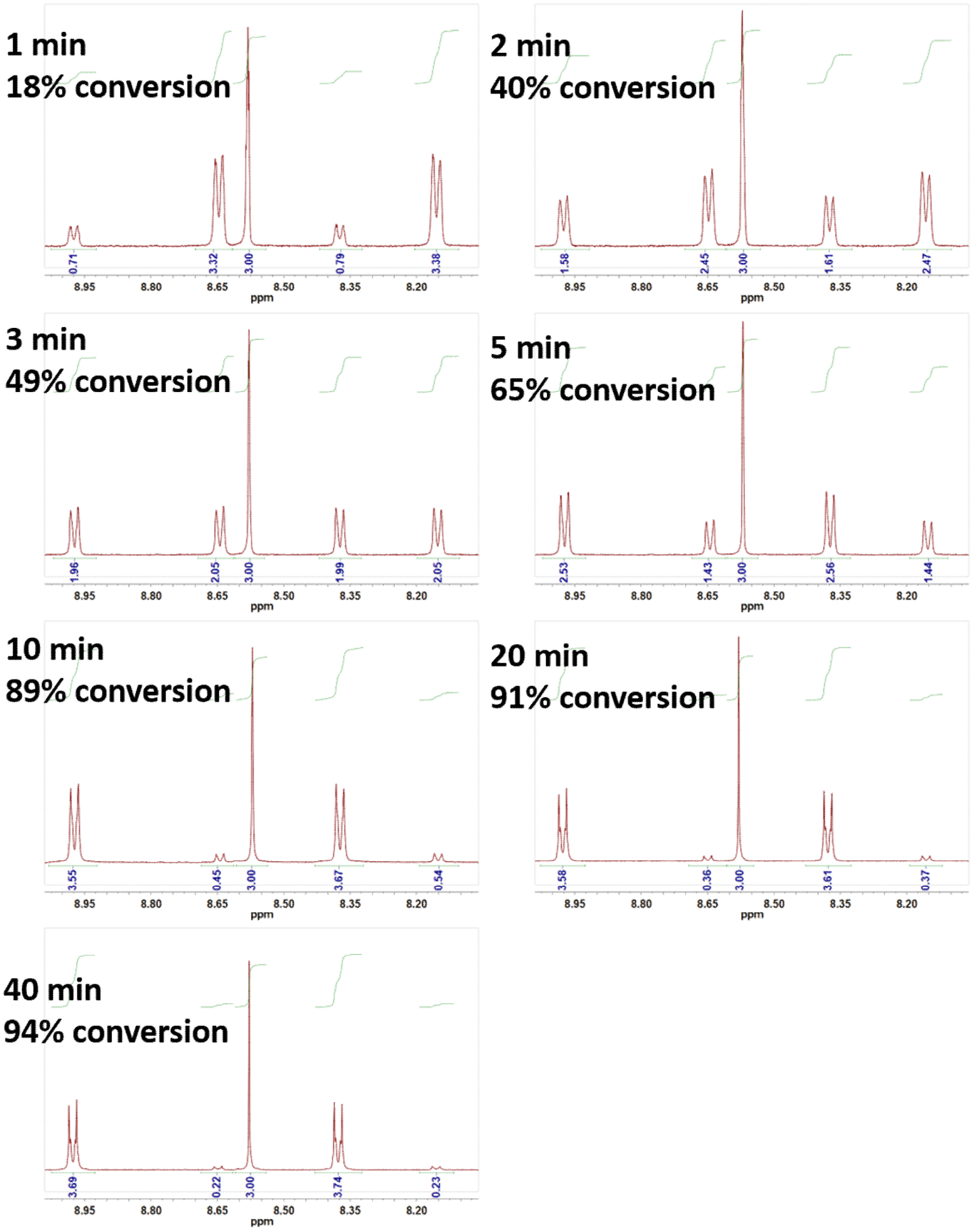

Supplementary Figure 11. ^{1}H NMR spectra of approximately 2 mg of well-ground HBE crystals soaked in 1.5 mL 0.3 M AP-DMF solution at 100 °C for given times.

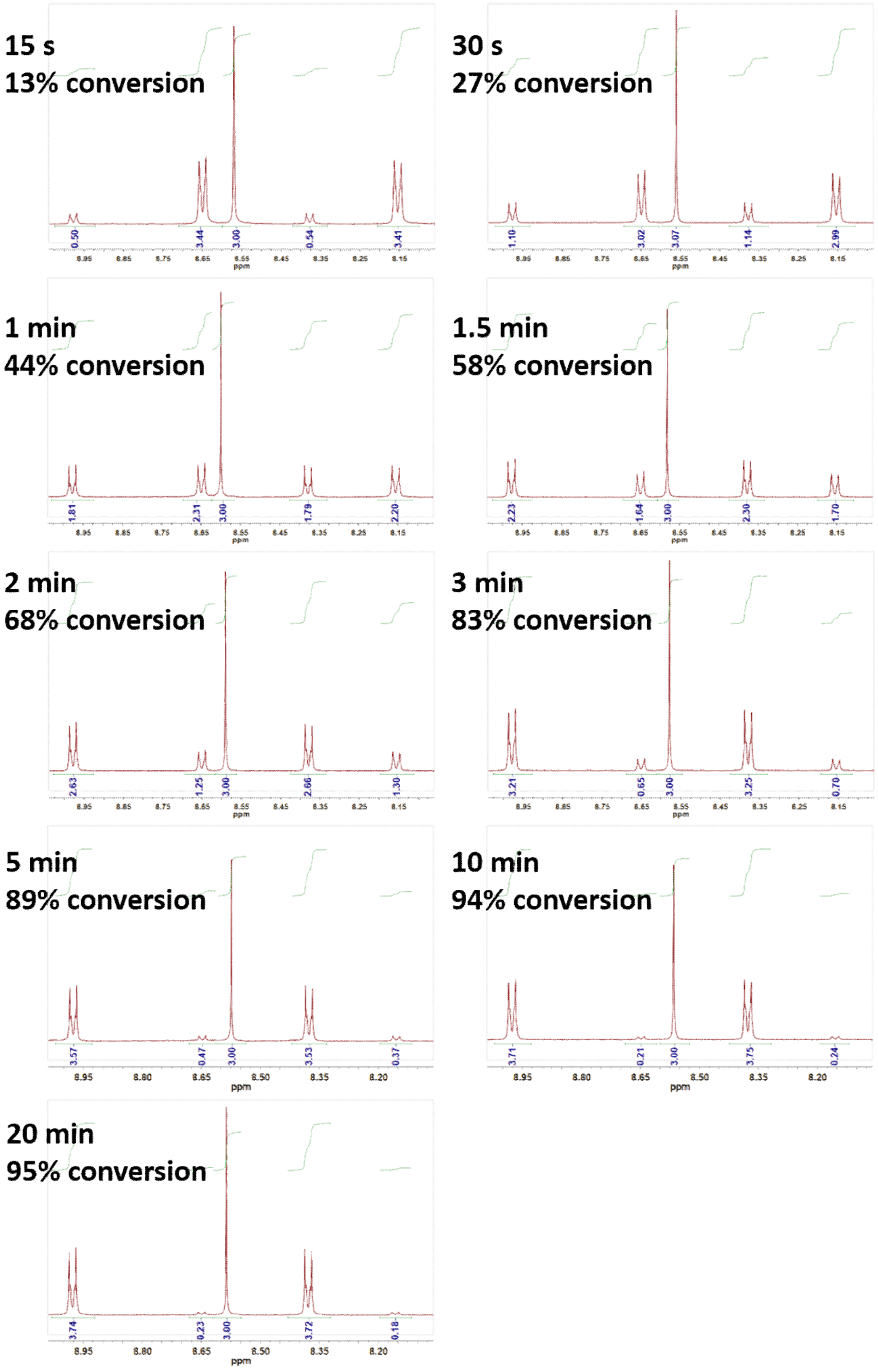

Supplementary Figure 12. ^{1}H NMR spectra of approximately 2 mg of well-ground HBE crystals soaked in 1.5 mL 0.3 M AP-DMF solution at 150 °C for given times.

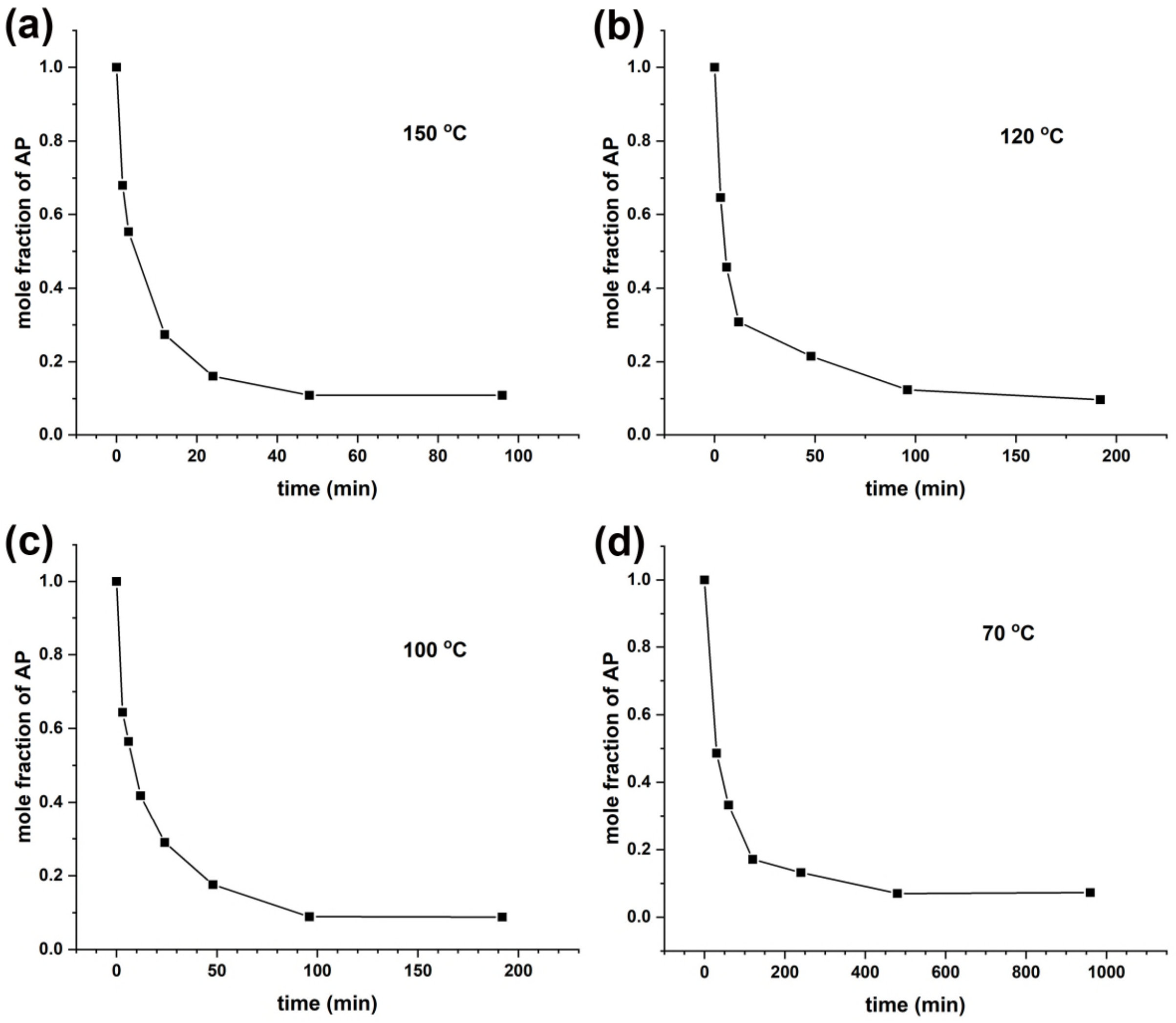

Supplementary Figure 13. Mole fraction of ligated AP pillars remaining in HAP2HBE crystals during the forward pillar exchange reactions of HAP crystals to HBE crystals at (a) 150 °C, (b) 120 °C, (c) 100 °C and (d) 70 °C, respectively. The mole fraction of ligated AP pillars at equilibrium conditions at a given temperature was chosen as the value at which there was no significant change in the mole fraction of ligated AP pillars with the additional reaction time.

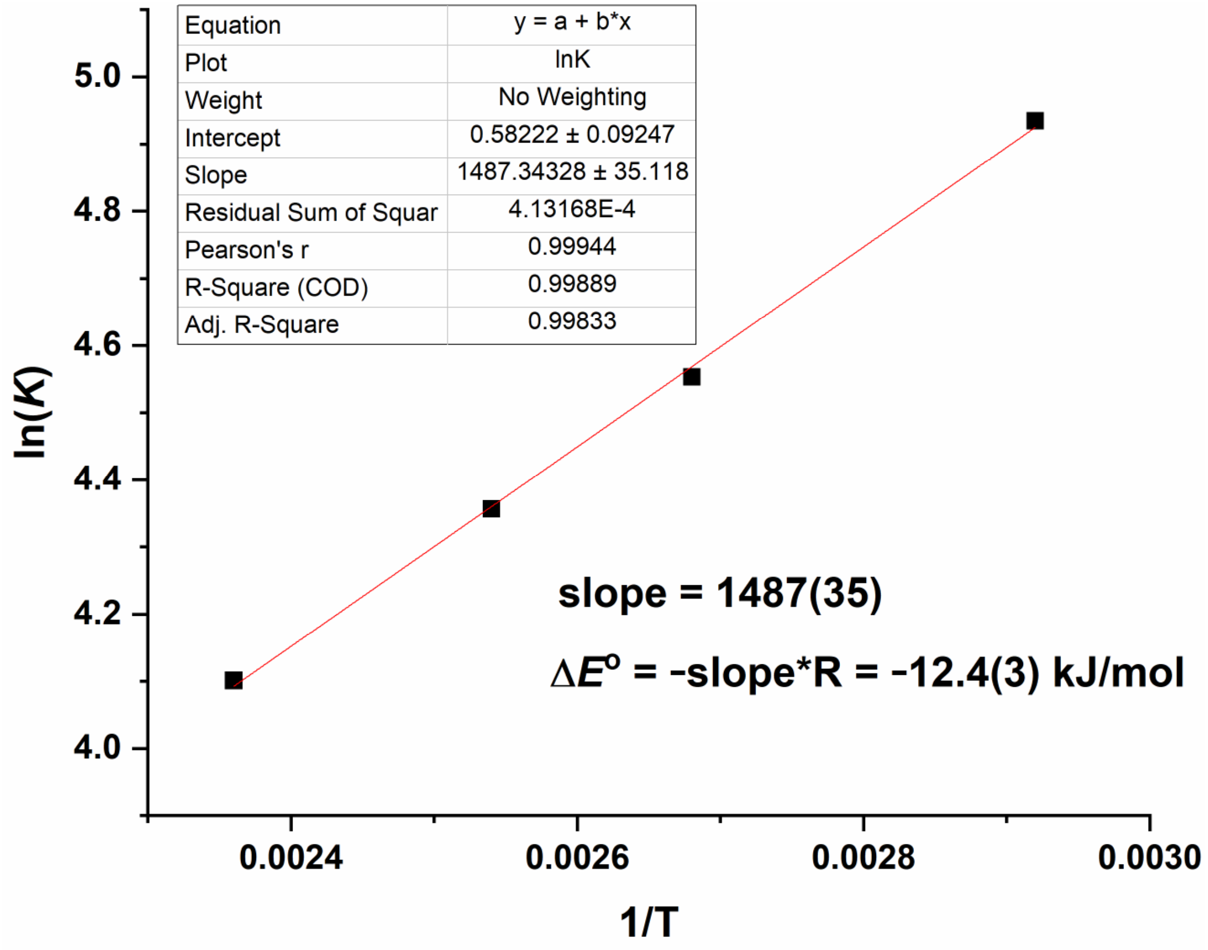

Supplementary Figure 14. Plot of ln(K) versus 1/T using temperature-dependent equilibrium constants over the temperature range of 50–150 °C, where the equilibrium constant K(T) at absolute temperature T is defined as [HBE][AP]/[HAP][BE] and absolute temperature T is defined as Celsius temperature t + 273. The concentration of ligated AP (or BE) pillars at equilibrium, [HAP] (or [HBE]), calculated from the mole fraction of ligated AP (or BE) pillars at equilibrium. The concentration of unligated AP (or BE) pillars was obtained as the initial concentration of ligated AP (or BE) pillars minus the equilibrium concentration of ligated AP (or BE) pillars.

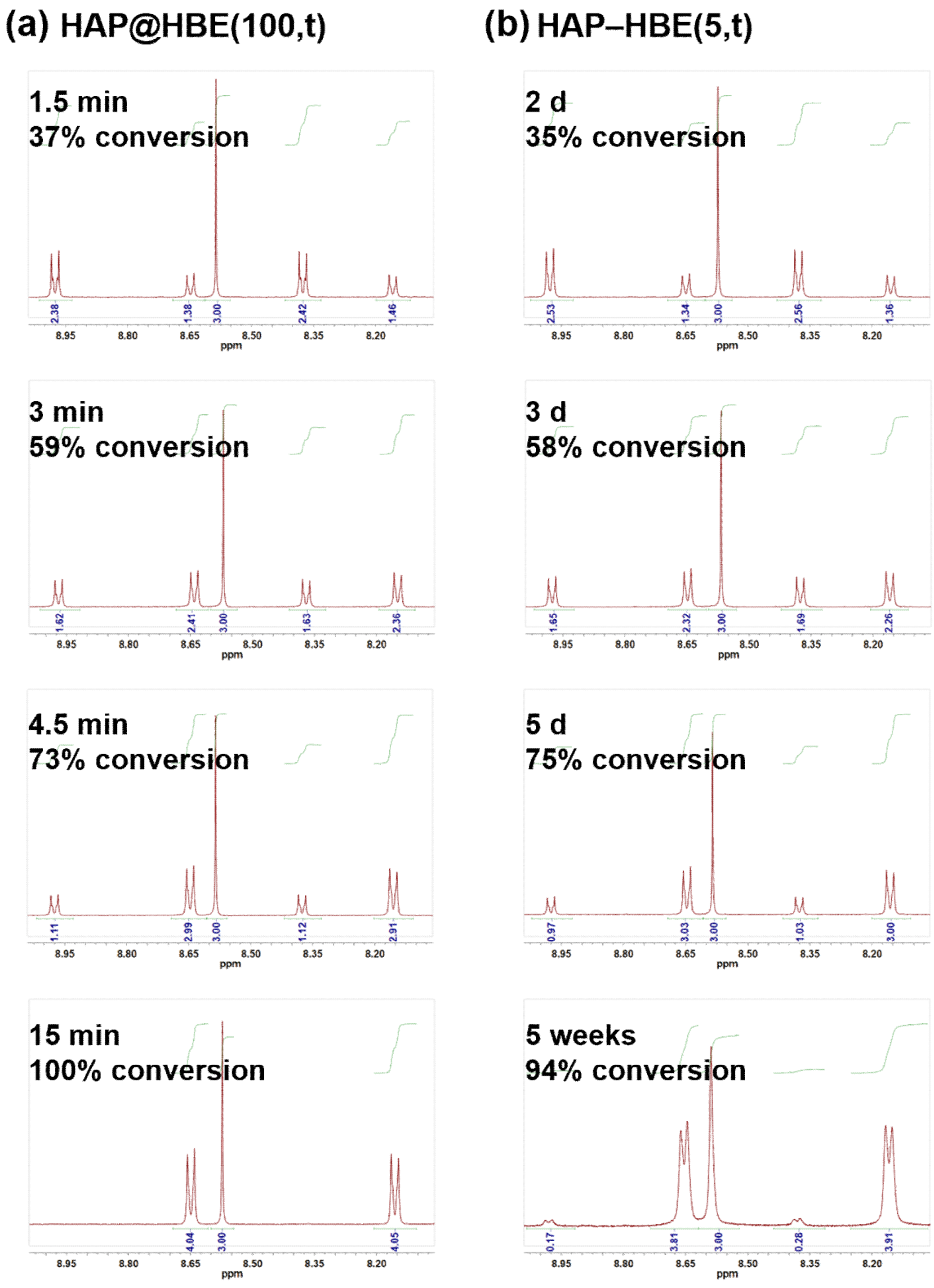

Supplementary Figure 15. ^{1}H NMR spectra of (a) core–shell microstructural intermediate, HAP@HBE(100,t), and (b) uniform microstructural intermediate, HAP–HBE(5,t), obtained by soaking ~2 mg of as-synthesized HAP crystals in 1.5 mL 0.3 M BE-DMF solution at 100 and 5 °C, respectively.

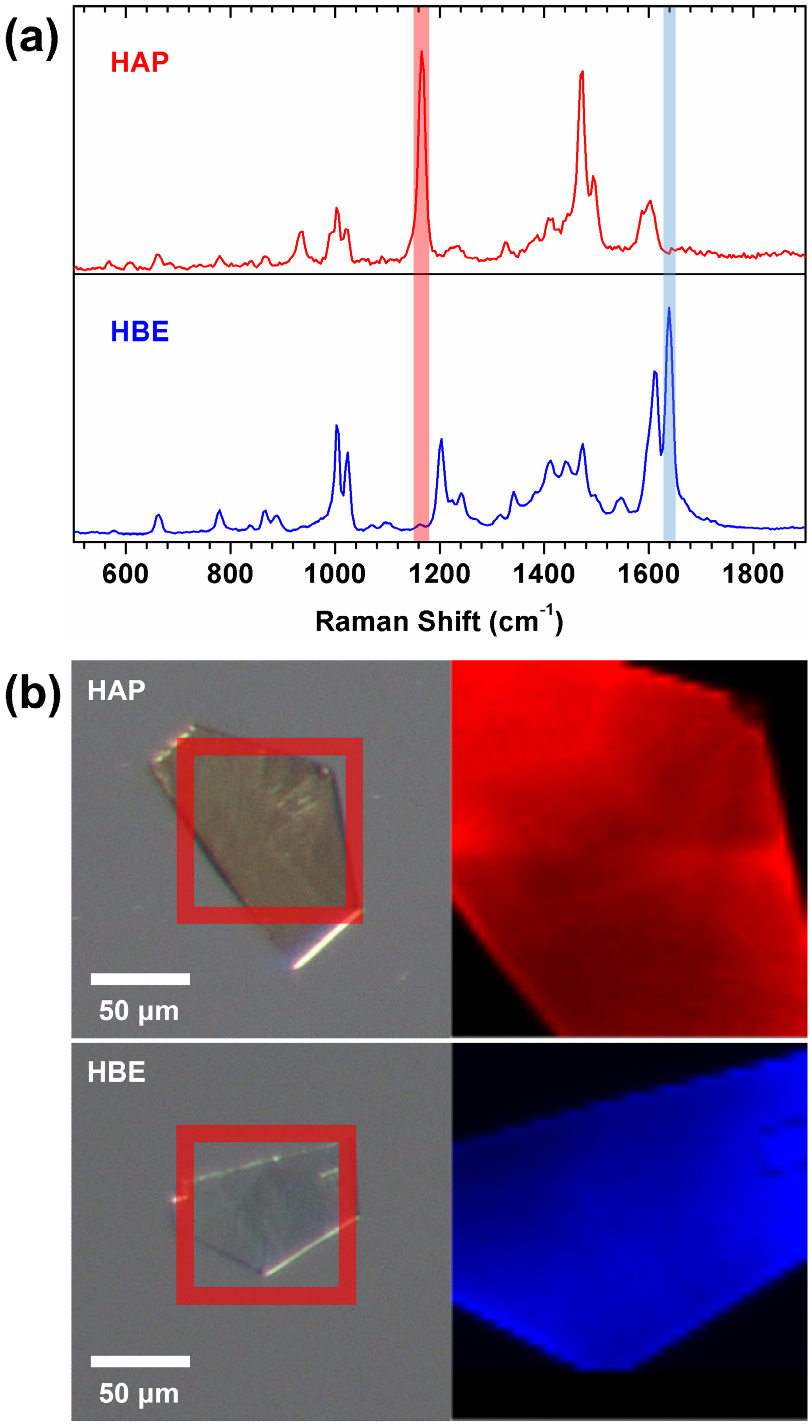

Supplementary Figure 16. (a) Raman spectra of HAP and HBE. The Raman bands at 1150–1180 cm^{-1} and 1630–1650 cm^{-1} were assigned to the AP pillars of HAP (decoded in red) and BE pillars of HBE (decoded in blue), respectively. (b) Optical photographs and Raman maps of the HAP and HBE single crystals.

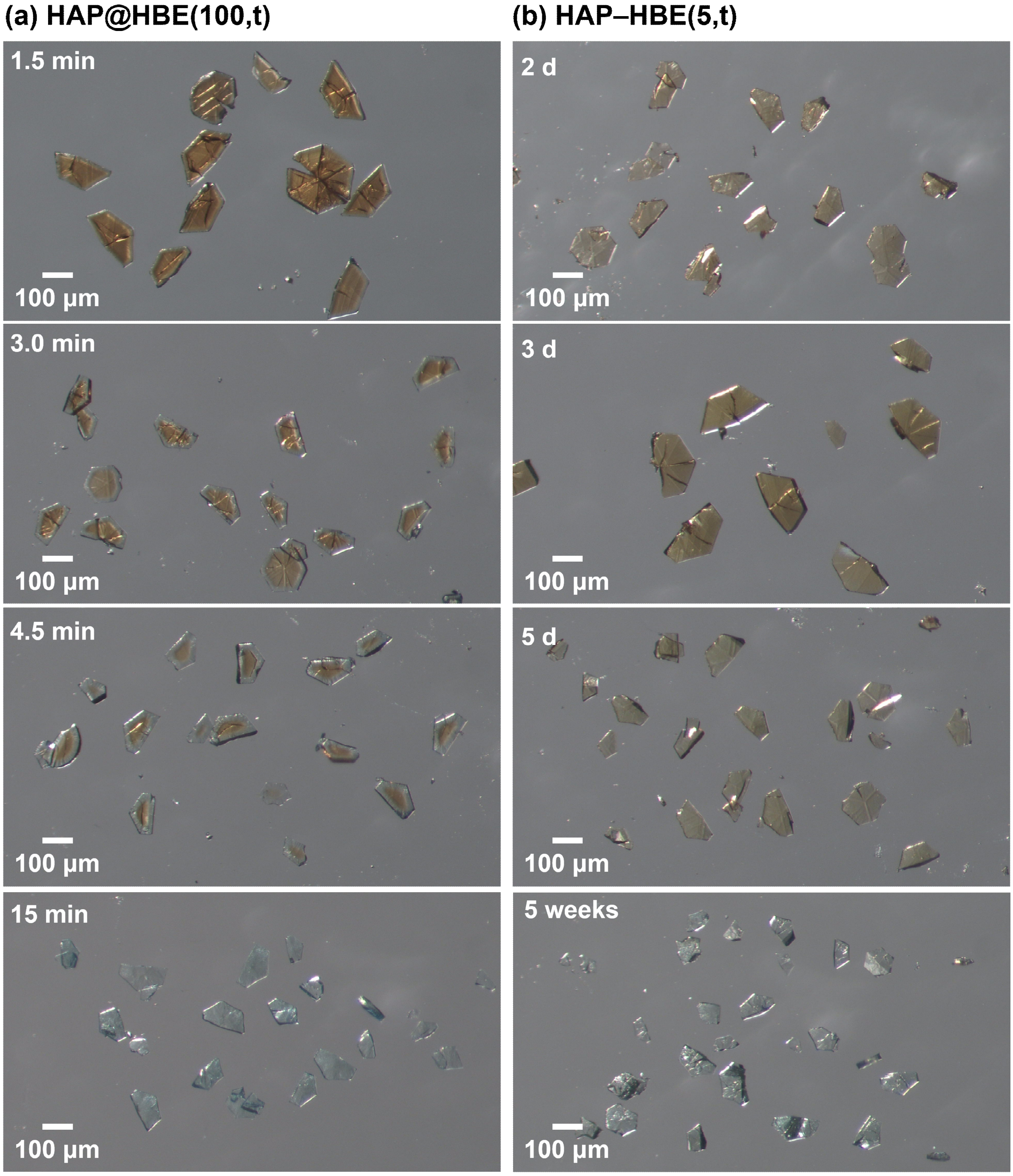

Supplementary Figure 17. Optical photographs of (a) concentric core–shell microstructural HAP@HBE(100,t) showing surface-to-core substitution of pillars and (b) uniform microstructural HAP–HBE(5,t) showing simultaneous substitution of pillars across the crystal.

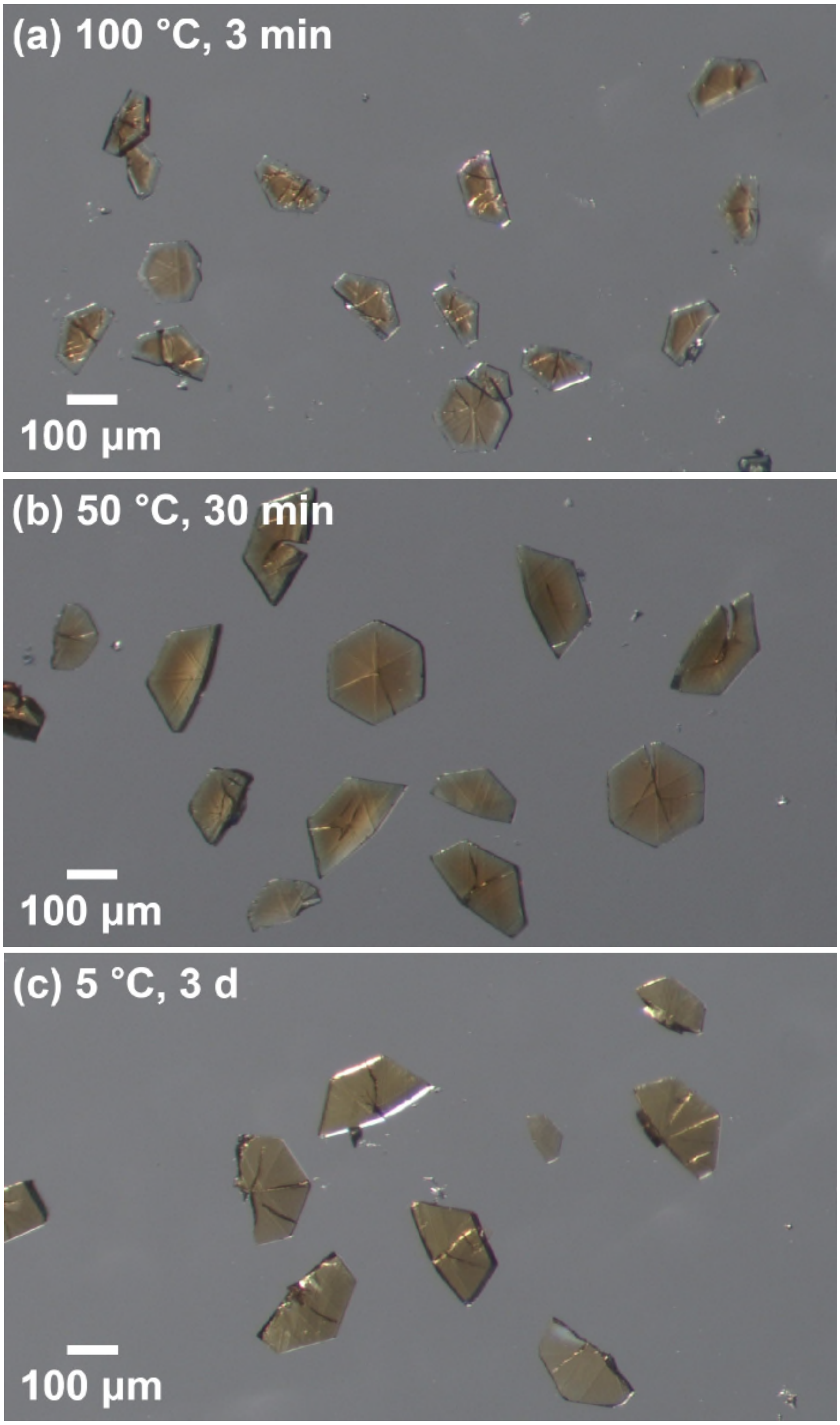

Supplementary Figure 18. Optical photographs of (a) HAP@HBE(100,3m), (b) HAP@HBE(50,30m), and (c) HAP–HBE(5,3d) with AP to BE pillar conversions of 59%, 62%, and 58%, respectively.

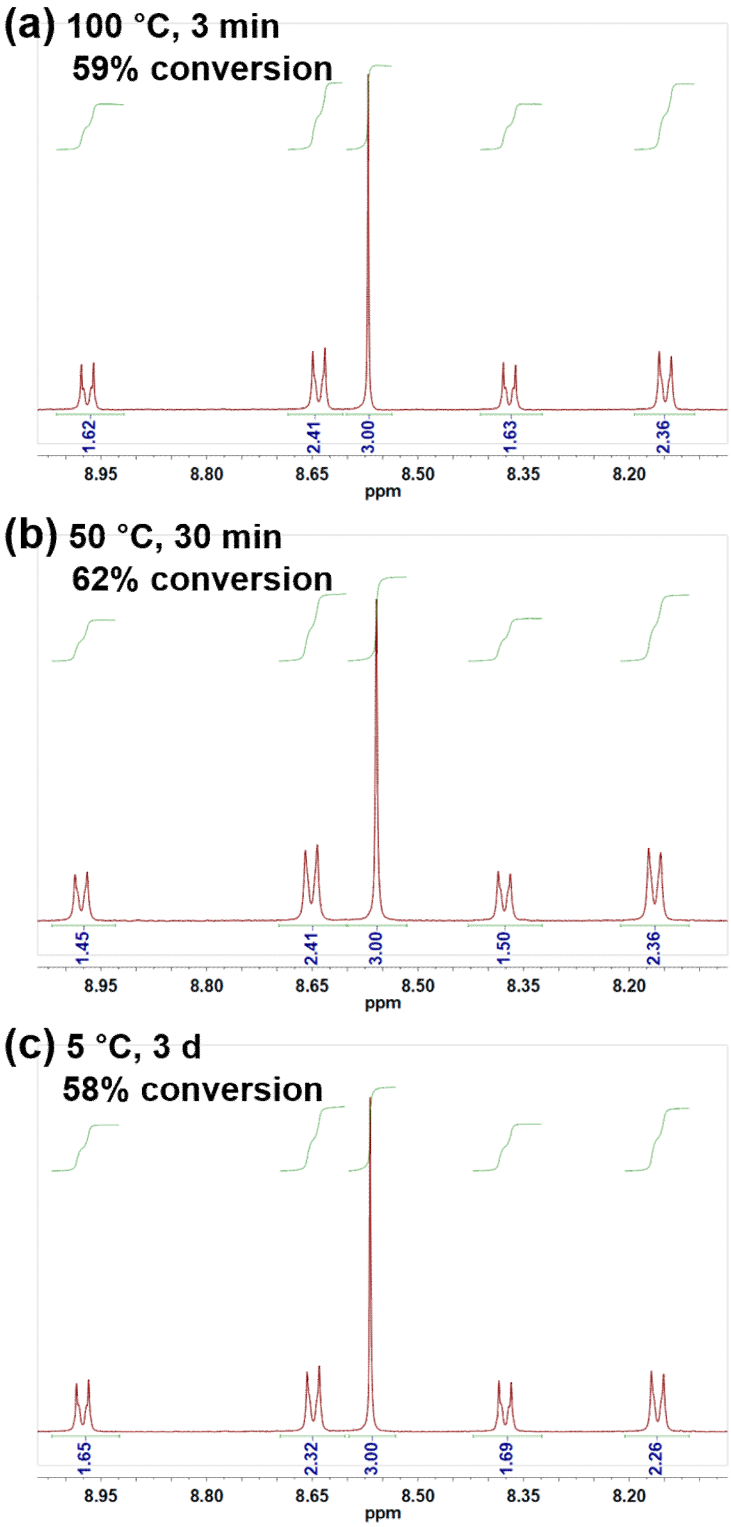

Supplementary Figure 19. ^{1}H NMR spectra of (a) HAP@HBE(100,3m), (b) HAP@HBE(50,30m), and (c) HAP–HBE(5,3d) with AP to BE pillar conversions of 59%, 62%, and 58%, respectively.

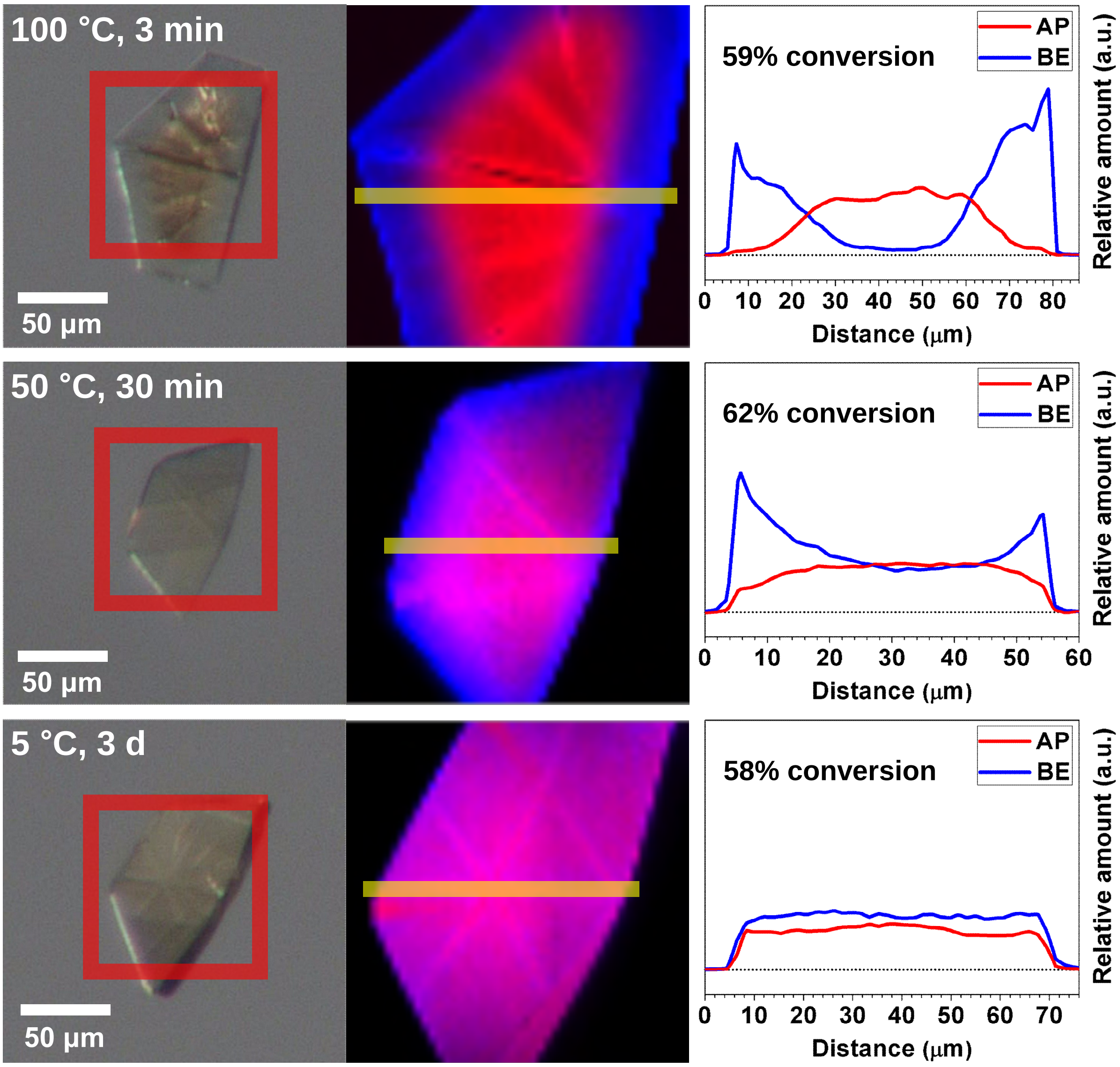

Supplementary Figure 20. Optical photographs, Raman maps, and relative amounts of AP and BE pillars showing a highly heterogeneous concentric core–shell pillar distribution in the intermediate, HAP@HBE$_{0.59}$(100,3m), a moderately heterogeneous concentric core–shell pillar distribution in the intermediate, HAP@HBE$_{0.62}$(50,30m), and a uniform pillar distribution in the intermediate, HAP–HBE$_{0.58}$(5,3d).

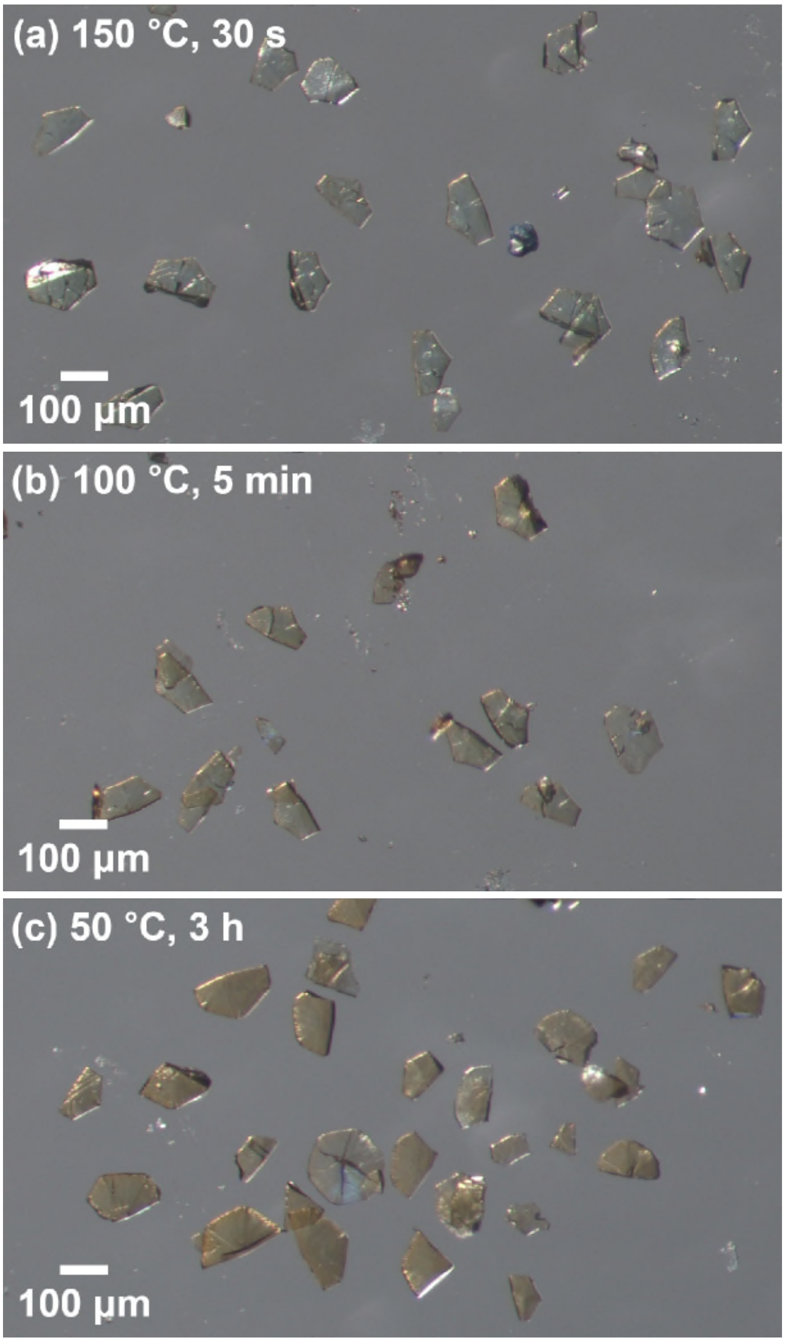

Supplementary Figure 21. Optical photographs of (a) HBE@HAP$_{0.31}$(150,30s) crystals showing a highly heterogeneous concentric inverted core–shell pillar distribution, (b) HBE@HAP$_{0.53}$(100,5m) crystals showing a moderately heterogeneous concentric inverted core–shell pillar distribution, and (c) HBE–HAP$_{0.48}$(50,3h) crystals showing a uniform pillar distribution.

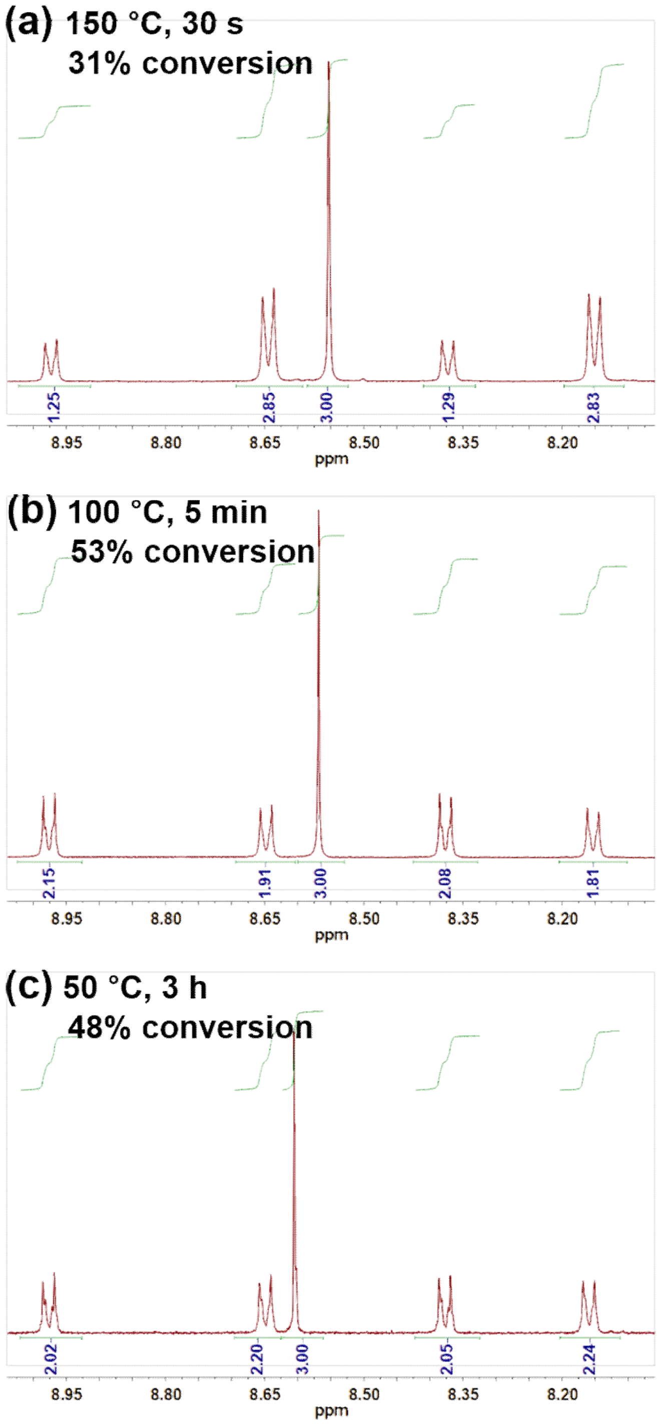

Supplementary Figure 22. ^{1}H-NMR spectra of (a) HBE@HAP(150,30s), (b) HBE@HAP(100,5m), and (c) HBE–HAP(50,3h) obtained by soaking ~2 mg of as-synthesized HBE crystals in 1.5 mL 1.0 M AP-DMF solution for 30 s at 150 °C, 5 min at 100 °C, and 3 h at 50 °C, respectively.

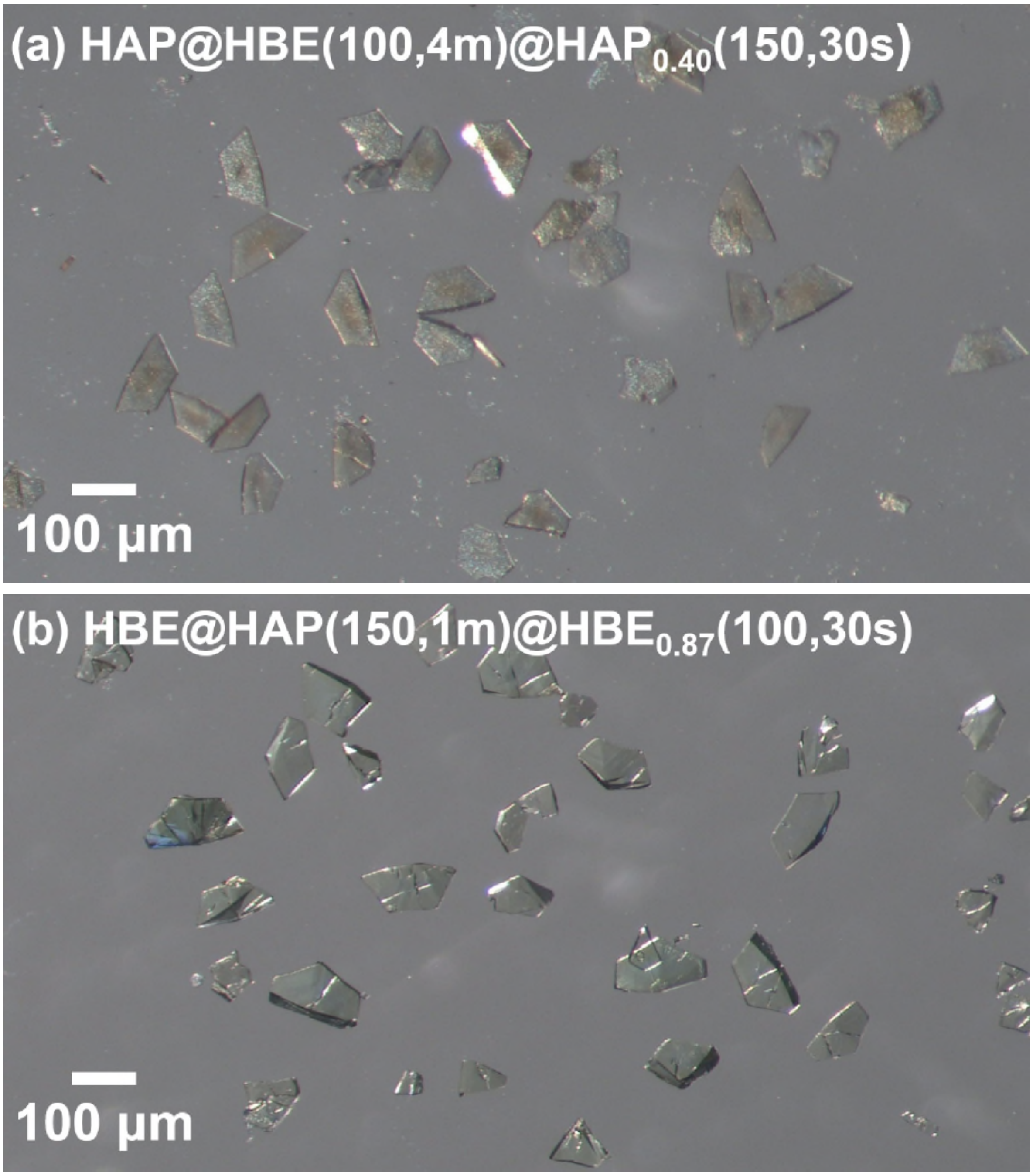

Supplementary Figure 23. Optical photographs of (a) HAP@HBE(100,4m)@HAP(150,30s) crystals showing concentric multi-shell pillar distribution and (b) HBE@HAP(150,1m)@HBE(100,30s) crystals showing concentric inverted multi-shell pillar distribution.

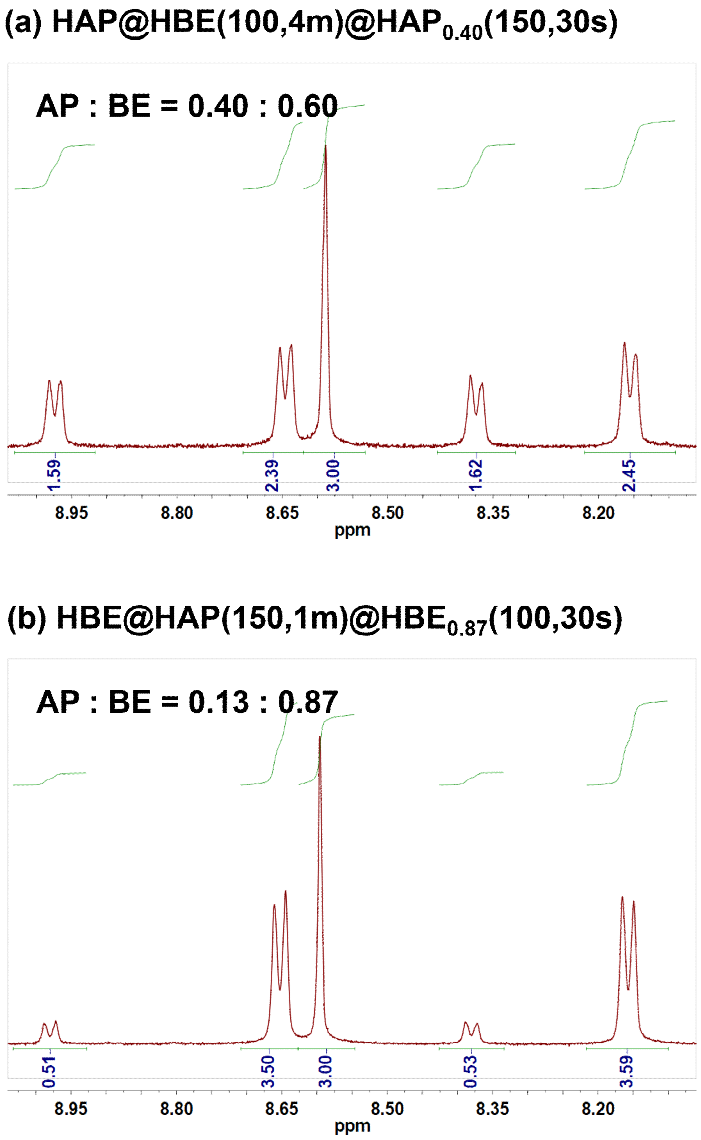

Supplementary Figure 24. ^{1}H-NMR spectra of (a) concentric inverted multi-shell microstructural HAP@HBE(100,4m)@HAP(150,30s) and (b) concentric inverted multi-shell microstructural HBE@HAP(150,1m)@HBE(100,30s).

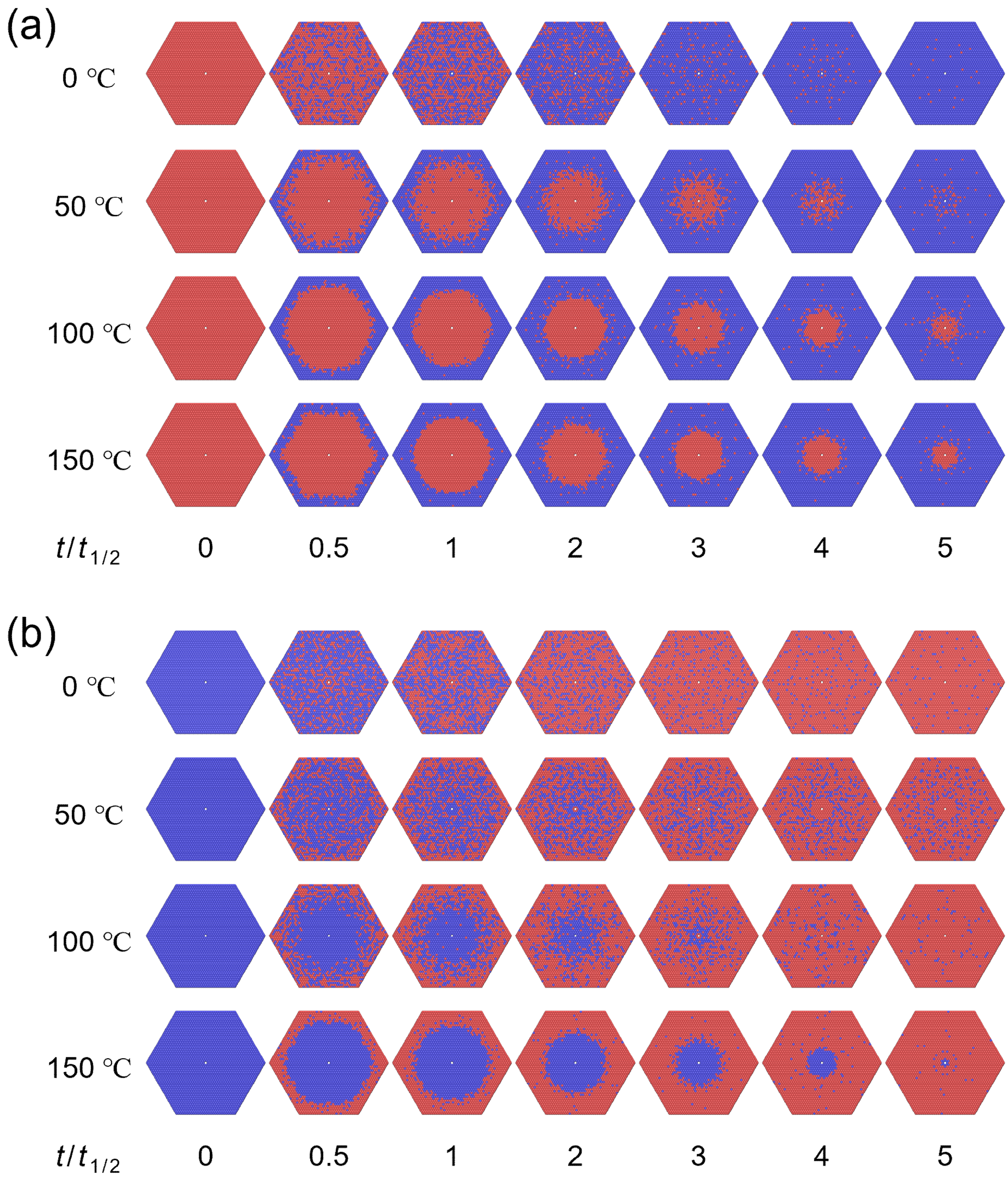

Supplementary Figure 25. Snapshots of spatial pillar distributions in hexagonal cells calculated using kMC simulations during (a) forward and (b) reverse exchange processes at different temperatures with ρ = 0.8. Red and blue dots represent the ligated pillars, AP and BE, respectively, and $t_{1/2}$ is the time required for 50% pillar exchange.

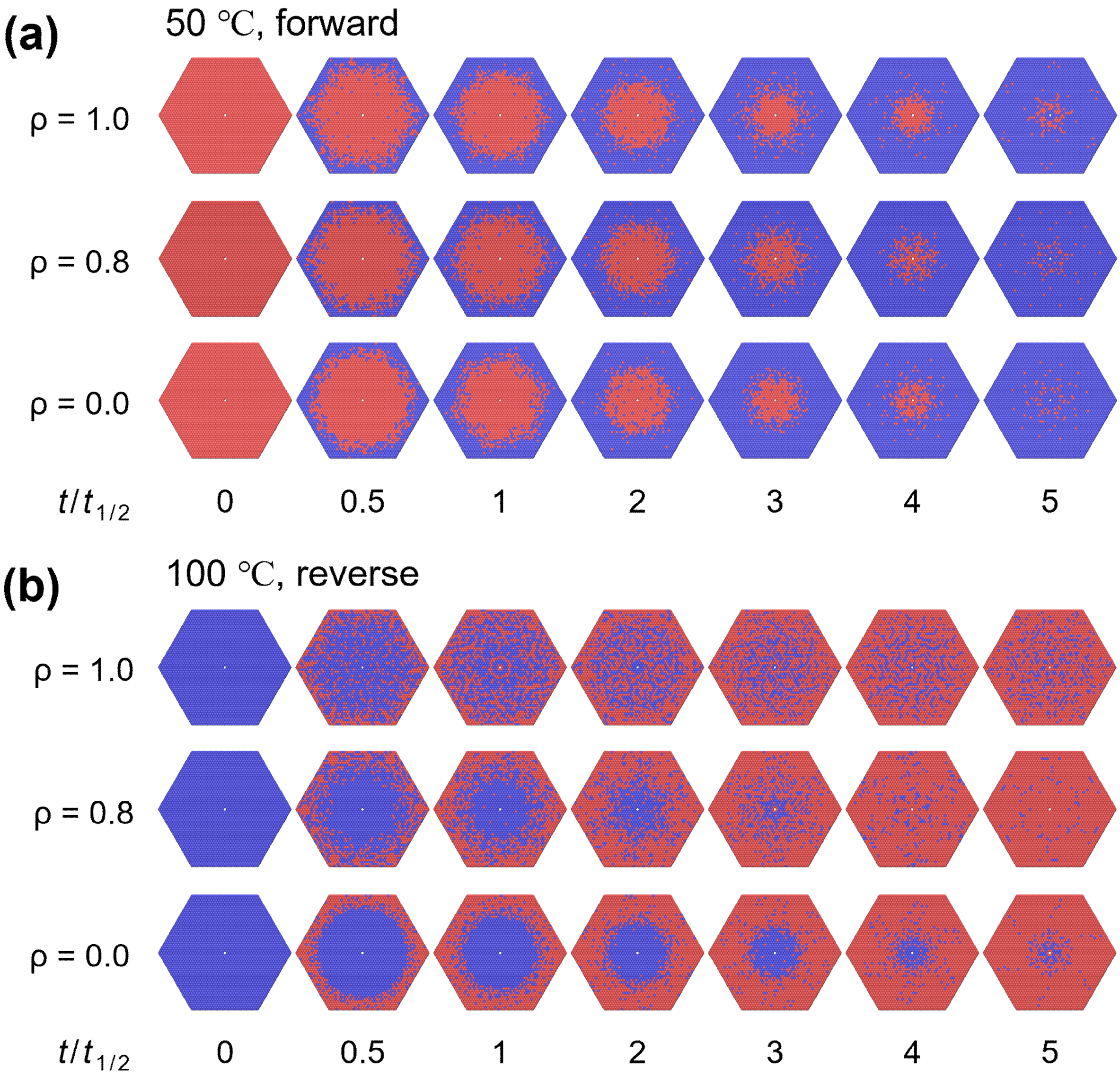

Supplementary Figure 26. Mean spatial distribution $D_{MAP/MBE}(x,t;T)$ of MAP and MBE in the hexagonal cell with different ρ values during (a) forward pillar exchange at 50 °C and (b) reverse pillar exchange at 100 °C.

Supplementary References

1. Kresse, G. & Furthmüller, J. Efficient iterative schemes for ab initio total-energy calculations using a plane-wave basis set. *Phys. Rev. B* **54**, 11169–11186 (1996).

2. Perdew, J. P., Burke, K. & Ernzerhof, M. Generalized gradient approximation made simple. *Phys. Rev. Lett.* **77**, 3865–3868 (1996).

3. Grimme, S., Antony, J., Ehrlich, S. & Krieg, H. A consistent and accurate ab initio parametrization of density functional dispersion correction (DFT-D) for the 94 elements H-Pu. *J. Chem. Phys.* **132**, 154104 (2010).

4. Jørgensen, M. & Grönbeck, H. Montecoffee: A programmable kinetic Monte Carlo framework. *J. Chem. Phys.* **149**, 114101 (2018).
